# Harmonizing the Collection of Clinical Data on Genetic Testing Requisition Forms to Enhance Variant Interpretation in Hypertrophic Cardiomyopathy (HCM): A Study from the ClinGen Cardiomyopathy Variant Curation Expert Panel

**DOI:** 10.1016/j.jmoldx.2021.01.014

**Published:** 2021-05

**Authors:** Ana Morales, Alexander Ing, Christian Antolik, Christina Austin-Tse, Linnea M. Baudhuin, Lucas Bronicki, Allison Cirino, Megan H. Hawley, Michael Fietz, John Garcia, Carolyn Ho, Jodie Ingles, Olga Jarinova, Tami Johnston, Melissa A. Kelly, C. Lisa Kurtz, Matt Lebo, Daniela Macaya, Lisa Mahanta, Joseph Maleszewski, Arjun K. Manrai, Mitzi Murray, Gabriele Richard, Chris Semsarian, Kate L. Thomson, Tom Winder, James S. Ware, Ray E. Hershberger, Birgit H. Funke, Matteo Vatta, ClinGen Cardiovascular Clinical Domain Working Group, Cardiomyopathy Variant Curation Expert Panel

**Affiliations:** ∗ Division of Human Genetics, The Ohio State University, Columbus, Ohio; † Invitae Corp., San Francisco, California; ‡ Laboratory for Molecular Medicine, Partners HealthCare, Boston, Massachusetts; § Department of Clinical Diagnostics, Ambry Genetics, Aliso Viejo, California; ¶ Department of Pathology, Massachusetts General Hospital, Boston, Massachusetts; ‖ Harvard Medical School, Boston, Massachusetts; ∗∗ Department of Laboratory Medicine and Pathology, Mayo Clinic, Rochester, Minnesota; †† Department of Genetics, CHEO, Ottawa, Ontario, Canada; ‡‡ Department of Pathology and Laboratory Medicine, University of Ottawa, Ottawa, Ontario, Canada; §§ Cardiovascular Division, Brigham and Women's Hospital, Boston, Massachusetts; ¶¶ PathWest Laboratory Medicine, Sir Charles Gairdner Hospital, Nedlands, Western Australia, Australia; ‖‖ Illumina Inc., Scoresby, Victoria, Australia; ∗∗∗ Cardiovascular Division, Departments of Medicine and Radiology, Brigham and Women's Hospital, Boston, Massachusetts; ††† Agnes Ginges Centre for Molecular Cardiology at Centenary Institute, The University of Sydney, Sydney, New South Wales, Australia; ‡‡‡ Geisinger, Danville, Pennsylvania; §§§ Department of Genetics, University of North Carolina, Chapel Hill, North Carolina; ¶¶¶ GeneDx, Inc, Gaithersburg, Maryland; ‖‖‖ Cardiovascular Medicine, Mayo Clinic, Rochester, Minnesota; ∗∗∗∗ Computational Health Informatics Program, Boston Children's Hospital and Harvard Medical School, Boston, Massachusetts; †††† Oxford Medical Genetics Laboratories, Churchill Hospital, Oxford, United Kingdom; ‡‡‡ National Heart and Lung Institute, Imperial College London, London, United Kingdom; §§§§ Cardiovascular Research Centre, Royal Brompton and Harefield NHS Foundation Trust, London, London, United Kingdom; ¶¶¶¶ MRC London Institute of Medical Sciences, Imperial College London, London, United Kingdom; ‖‖‖‖ Division of Cardiovascular Medicine, The Ohio State University, Columbus, Ohio; ∗∗∗∗∗ Departments of Medical and Molecular Genetics, Indiana University School of Medicine, Indianapolis, Indiana

## Abstract

Diagnostic laboratories gather phenotypic data through requisition forms, but there is no consensus as to which data are essential for variant interpretation. The ClinGen Cardiomyopathy Variant Curation Expert Panel defined a phenotypic data set for hypertrophic cardiomyopathy (HCM) variant interpretation, with the goal of standardizing requisition forms. Phenotypic data elements listed on requisition forms from nine leading cardiomyopathy testing laboratories were compiled to assess divergence in data collection. A pilot of 50 HCM cases was implemented to determine the feasibility of harmonizing data collection. Laboratory directors were surveyed to gauge potential for adoption of a minimal data set. Wide divergence was observed in the phenotypic data fields in requisition forms. The 50-case pilot showed that although demographics and assertion of a clinical diagnosis of HCM had 86% to 98% completion, specific phenotypic features, such as degree of left ventricular hypertrophy, ejection fraction, and suspected syndromic disease, were completed only 24% to 44% of the time. Nine data elements were deemed essential for variant classification by the expert panel. Participating laboratories unanimously expressed a willingness to adopt these data elements in their requisition forms. This study demonstrates the value of comparing and sharing best practices through an expert group, such as the ClinGen Program, to enhance variant interpretation, providing a foundation for leveraging cumulative case-level data in public databases and ultimately improving patient care.

Patient phenotype is a common field on laboratory requisition forms. As an item of the College of American Pathologists checklist for laboratory results classification (*https://elss.cap.org/elss/ShowProperty?nodePath=/UCMCON/Contribution%20Folders/DctmContent/education/OnlineCourseContent/2017/LAP-TLTM/checklists/cl-mol.pdf*, last accessed August 7, 2020), a patient's phenotypic data not only may be taken into consideration when providing a clinical interpretation of a result by the health care provider, but they also may aid in variant classification.^1^ This has become increasingly critical with the continued expansion of genetic testing, resulting in ever-growing volumes of genomic data to analyze. Guidelines from the American College of Medical Genetics and Genomics and the Association for Molecular Pathology on sequence variant classification^2^ emphasize the importance of case-level data for phenotype-supported proband (PS4 and PP4), segregation (PP1), and *de novo* occurrence criteria (PS2 and PM6) that are used in variant interpretation. This is illustrated by *MYH7 p.Ala1379Thr*, which would have remained a variant of uncertain significance without the availability of phenotype-based case data,^3^, ^4^, ^5^, ^6^, ^7^ including segregation with hypertrophic cardiomyopathy (HCM) across three families with a total of 15 affected relatives.^8^

As medical genetics is increasingly incorporated into routine clinical practice, diagnostic laboratories have been able to obtain phenotypic data from ordering providers and are in a position to be major contributors to variant knowledge because of the large number of cases that are processed. Wain et al^9^ emphasized this concept, highlighting the importance of submitting phenotype information to laboratories. The utility of harmonized case-level phenotypic data is twofold:

(1) Outside of rare disease and whole exome/genome testing, laboratories typically do not need extensive phenotypic data for processing a specific case (as the emphasis is not to diagnose a patient, but rather to provide an interpretation of sequence findings). However, certain phenotypic data elements can prove helpful in refining the clinical interpretation of variants. Variant classification in HCM requires phenotype and family history information, such as documentation of syndromic features, evidence of physiological remodeling, absence of family history of HCM,^10^ or increased disease severity (which may be due to multiple variants) that is not usually obtained to establish a clinical diagnosis. Given these complexities, a simple check mark in a box on a requisition form indicating a diagnosis of HCM is often insufficient.
(2) Similar to aggregating published case-level data, it is increasingly recognized that combining unpublished case data internal to each laboratory across laboratories can significantly improve variant classification.^11^^,^^12^ However, although laboratories have been successful at collecting phenotypic data, they have operated in isolation without consensus standards on what information is most critical, hampering their ability to harness the power of aggregate data.

In the case of HCM, clinical diagnostic criteria are published. Both the American Heart Association/American College of Cardiology^13^ and the European Society of Cardiology^14^ guidelines state that HCM is clinically defined as a wall thickness of ≥15 mm, recognizing that thickness of 13 to 14 mm may be present in the setting of familial disease or a genetic predisposition. However, differences exist among these guidelines. Although the European Society of Cardiology definition of HCM includes left ventricular hypertrophy (LVH) in the absence of a hemodynamic cause, the American Heart Association/American College of Cardiology definition also requires exclusion of other cardiac or systemic disease. In addition, the community has not yet assessed the amount of clinical information that is useful. The lack of consensus on what phenotypic data are considered necessary and sufficient for adequately interpreting genetic testing results has not been documented in the literature. Led by ClinGen's Cardiomyopathy Variant Curation Expert Panel, herein referred to as the ClinGen Expert Panel, this study was set out to determine the minimum phenotypic data elements required to assign affected status for a case during variant classification and enhance the ability to interpret complex cases during variant classification for HCM.

## Materials and Methods

### Development of the Expert Panel Cardiovascular Phenotypic Data Elements List

Members of the expert panel developed a list of cardiovascular data elements typically documented in cardiology clinic notes and included in requisition forms. The list, which was generated to define the data collection approach for this study, was drafted by a cardiovascular genetic counselor (A.M.), and revisions were made by cardiologists (R.E.H., C.S., and J.W.) to ensure that the list was representative of the standard clinical diagnostic criteria of HCM (Table 1). This comprehensive list was based on existing requisition forms and input from expert HCM clinicians. However, the list was not intended to replace or redefine the clinical diagnostic criteria for HCM, which should be determined only by physician expertise. It was rather intended to identify individuals to be counted as bona fide cases, consistent with the PS4 and PP1 evidence from the 2015 American College of Medical Genetics and Genomics/Association for Molecular Pathology guidelines.^2^

**Table 1:** Data Elements Proposed by the Expert Panel

Expert panel cardiovascular data elements list	Information provided (LMM pilot study)	Participating laboratories reported frequency of obtaining data, %			Final consensus
		Very frequently and frequently	Sometimes	Very infrequently and infrequently	Data elements deemed essential for variant interpretation
Sex	48/50	100	0	0	Y
Race and ethnicity	43/50	50	25	25	Y
Current age	50/50	100	0	0	Y
Family history	45/50	62.5	25	12.5	Y
Clinical diagnosis of HCM	49/50	62.5	37.5	0	Y
Age at diagnosis	25/50	12.5	62.5	25	Y
Left ventricular hypertrophy	22/50	25	75	0	Y
Left ventricular hypertrophy measurement	15/50	25	25	50	Y
Left ventricular outflow tract obstruction	0/50	0	71.43	28.58	N
Reduced ejection fraction	12/50	0	62.5	37.5	N
Ejection fraction percentage	2/50	12.5	25	62.5	N
History of hypertension	16/50	0	50	50	Y
Blood pressure on treatment	0/50	14.29	14.29	71.43	Y
Suspected syndromic HCM/other cause	15/50	0	0	100	Y
ECG with left ventricular hypertrophy or atrial fibrillation	22/50	0	25	75	N
History of syncope	0/50	14.29	57.14	28.57	N
Nonsustained ventricular tachycardia on Holter	3/50	0	12.5	87.5	N
Late gadolinium enhancement on cardiac MRI	0/50	0	0	100	N

ECG, electrocardiogram; HCM, hypertrophic cardiomyopathy; LMM, Laboratory for Molecular Medicine; MRI, magnetic resonance imaging; N, no; Y, yes.

### Review of Existing Laboratory Requisition Forms

To characterize the extent of discrepancy across laboratory requisition forms used in the community, review of the Genetic Testing Registry database (*https://www.ncbi.nlm.nih.gov/gtr*, last accessed August 10, 2016) was performed for laboratories offering DNA-based testing for HCM. The requisition forms were downloaded from their respective websites, and each field was documented. Data were aggregated to identify fields that were common across laboratories. If fields requesting similar data but using different descriptors were identified, one term was chosen.

### Retrospective Case Requisition Review

A pilot study was designed to examine provider compliance in providing data on requisition forms and to determine the feasibility of harmonizing laboratory requisition forms based on the expert panel–derived phenotypic data elements. The pilot study consisted of 50 consecutive cases sent for HCM testing to the Laboratory for Molecular Medicine (LMM; a representative laboratory) between June 2015 and June 2016. The requisition fields on the LMM form were matched to the proposed expert panel data elements list. If a field was completed by the ordering provider, it was counted as completed, independently of the answer.

### Laboratory Director Survey Development

Using insights gained from the retrospective case requisition review, a Laboratory Director Survey was developed by the study principals (A.M., A.I., and M.V.) and conducted in the spring of 2018 to determine whether the proposed criteria were essential and sufficient for variant classification (Supplemental Appendix S1).

### Laboratory Director Survey: Participant Recruitment

Survey participants were recruited from laboratories offering cardiomyopathy genetic testing that also used expert panel members or associates. An introductory e-mail was sent, inviting all laboratory directors to participate and to provide one response per laboratory. Confidentiality was assured, and participants were informed that only aggregate data would be reported. The senior author (M.V.), a clinical molecular geneticist acting as a laboratory director signing out cardiovascular genetic reports, designated an alternate to complete the survey.

### Expert Panel Conflict of Interest Management

Before this study, and as part of their initial recruitment to the expert panel, participants were required to declare conflicts of interest. Financial conflict of interest was defined as having a financial relationship with a commercial entity that provides genetic testing services or where there could be a vested interest in a particular gene and variant classification. Individuals may be perceived to have an academic conflict of interest when they have participated in scientific discoveries or the general body of knowledge regarding a particular gene or variant.

### Consensus Building

The consensus building phase consisted of surveys and telephone calls. On the basis of the results of the Laboratory Director Survey, a second survey was designed by study principals (ClinGen Expert Panel Survey). It contained five questions, including one question in which endorsement of the proposed criteria was assessed (Supplemental Appendix S2) and was sent to members of the ClinGen Expert Panel on December 19, 2018, and closed on January 18, 2019. The members invited to participate were informed that lack of a response was interpreted as acceptance of the proposal. Majority approval was defined as obtaining support from at least three-fourths of the members. Survey results were discussed during ClinGen Expert Panel follow-up calls, at which time consensus was reached.

## Results

### Review of Existing Laboratory Requisition Forms

A total of 63 data elements were represented across requisition forms from nine laboratories (Table 2). Comparison of structured fields showed that the criteria on the expert panel–derived list were not consistently identified across laboratories. Furthermore, there were substantial differences in the types of phenotypic data requested by laboratories.

**Table 2:** Data Collection on Requisition Forms in Selected Laboratories

Variable	Laboratory 1	Laboratory 2	Laboratory 3	Laboratory 4	Laboratory 5	Laboratory 6	Laboratory 7	Laboratory 8	Laboratory 9
Demographics									
Sex	X	X	X	X	X	X	X		X
Ethnicity/ancestry	X	X	X	X	X	X	X		X
Family history									
Syncope					X				
Episodes					X				
Cardiac arrest/sudden cardiac death					X		X	X	
HCM							X	X	
Congestive cardiac failure								X	
Stroke									
Other cardiomyopathy							X		
Family genetic testing		X							
Free text (for family history only)	X	X	X	X	X	X	X	X	X
Proband history (risk factors)									
Sudden cardiac arrest history Y/N	X	X		X			X		
If SCD, number of episodes	X								
If SCD, age at first episode	X								
Hypertension		X							
History of syncope Y/N	X	X		X					
Other symptoms	X	X		X			X		X
Proband history (named diagnoses)									
Free text (summary field for all clinical information)	X	X	X	X	X	X	X	X	X
Unknown diagnosis		X					X		X
Unaffected				X			X		X
Age at diagnosis	X	X		X			X		
Cardiomyopathy diagnosis Y/N	X	X		X			X		X
HCM	X	X		X			X	X	X
Conventional diagnostic criteria for HCM								X	
Other cardiomyopathy	X	X		X			X		X
Arrhythmia diagnosis Y/N	X	X		X			X		X
Atrial fibrillation		X		X			X		X
Ventricular tachycardia		X		X			X		X
WPW		X					X		X
Other arrhythmia types	X	X		X			X		X
Features of Danon							X		
Features of Fabry							X		
Other genetic conditions	X						X		
Previous genetic testing	X	X				X			
Cardiac procedure questions									
Procedures, Y/N (eg, ECG or ECHO)	X	X		X			X		
Procedure (eg, ECG or ECHO) age	X								
Procedure result (eg, LVIDd, PWd, or Qtc)	X	X		X					
ECG	X	X		X			X	X	
Other (ECG)									
Free text				X					
ECHO	X	X		X			X	X	
Ejection fraction, %				X	X		X		X
Ventricular hypertrophy		X					X		X
Left		X							
Right		X							
Max LV wall thickness		X		X	X		X	X	X
Asymmetric							X	X	X
Concentric							X		X
SAM of mitral valve								X	
Diastolic dysfunction								X	
Cardiac MRI		X					X		
Cardiac MRI maximum LV wall thickness		X			X				
Other procedures									
Histology								X	
Myocardial disarray								X	
Heart transplant				X					
Cardiovascular device implant (eg, pacemaker, ICD, or LVAD)	X	X			X				
Age at implantation	X								
Device type	X	X			X				
Pacemaker	X	X							
ICD	X	X							
LVAD	X	X							
Other procedure	X	X							
Additional history	X								

ECG, electrocardiogram; ECHO, echocardiogram; HCM, hypertrophic cardiomyopathy; ICD, implantable cardioverter-defibrillator; LV, left ventricular; LVAD, LV assist device; MRI, magnetic resonance imaging; N, no; PWd, P-wave dispersion; SCD, sudden cardiac death; WPW, Wolff-Parkinson-White syndrome; Y, yes.

Most broad categories, such as demographics and family history, were present on most laboratories' requisition forms. The specificity of the family history field, however, varied among laboratories. For example, some laboratories asked specific cardiomyopathy questions, including cardiomyopathy type or symptoms in the family, whereas others only asked a general question for the ordering provider to complete in free text form. Similarly, six laboratories requested information specifically regarding the patient's cardiomyopathy, although no specific phenotype features were consistently identified as a data element field across all nine forms (eg, diagnosis of cardiomyopathy, Y/N? or left ventricular measurement).

### Retrospective Case Requisition Review

Review of the LMM requisition form used for the pilot study of 50 HCM cases revealed that, with the exception of left ventricular outflow tract obstruction, blood pressure on treatment, history of syncope, and late gadolinium enhancement on cardiac magnetic resonance imaging, all of the initial elements on the expert panel phenotypic data list were represented by a stand-alone unique field (Table 1). Common demographics and basic phenotype fields were completed most of the time for the 50 cases, including sex (48/50; 96%), race and ethnicity (43/50; 86%), clinical diagnosis of HCM (49/50; 98%), and family history of HCM (45/50; 90%). However, specific phenotype features were completed less frequently, including LVH (22/50; 44%), reduced ejection fraction (12/50; 24%), and suspected syndromic HCM/other cause (15/50; 30%). Of note, two of the evaluated cases were specifically suspected to have a syndromic cause of HCM, suggesting that some of the cases with a reported clinical diagnosis of HCM may instead have had LVH due to a multisystem genetic etiology, and genetic testing was ordered to confirm the diagnosis. Data from this pilot support that most phenotypic data fields on requisition forms are not completed, and ordering providers generally list only limited phenotype information.

### Laboratory Director Survey and Refinement of Essential Phenotypic Data Elements

Ten laboratories were invited, of which eight completed the survey. Participants included five US laboratories (Laboratory for Molecular Medicine; Ambry Genetics; GeneDx; Invitae; and Mayo Clinic) and three international laboratories (Children's Hospital of Eastern Ontario, Canada; Oxford Molecular Genetics Laboratory, UK; and PathWest Laboratory Medicine, Australia). One response per laboratory was allowed. The participating laboratories are not necessarily the same laboratories represented in Table 2.

Participants were asked to evaluate the frequency of expert panel–derived phenotypic data elements received via clinical testing requisition forms. Similar to the 50-case pilot using LMM cases, demographic information (sex, race and ethnicity, and family history) and indication of a clinical diagnosis of HCM were received very frequently or frequently, whereas the age of diagnosis and presence of LVH or hypertension were noted sometimes, and nuanced clinical information, such as LVH measurement or imaging, was infrequently or very infrequently provided. The remainder of the data elements were only infrequently or very infrequently obtained.

The survey data did not explicitly identify criteria in order of importance; however, on the basis of participants' judgment, presence of left ventricular outflow tract obstruction, reduced ejection fraction (and percentage), electrocardiogram with LVH or atrial fibrillation, history of syncope, nonsustained ventricular tachycardia on Holter monitoring, and late gadolinium enhancement on cardiac magnetic resonance imaging were deemed nonessential for variant interpretation, considering that these data may not be sufficient to establish a case of HCM that could be added to the evidence base of a given variant. The survey resulted in 11 criteria, representing the minimum key clinical data elements recommended by laboratory directors for standard inclusion in requisition forms for HCM variant interpretation (Table 1).

### Considerations on Practicality and Feasibility of Implementation

All eight laboratory director participants agreed with standardizing the phenotypic data elements and future implementation. They also raised questions about practical feasibility and whether all proposed elements were essential components in their variant classification workflows. Five laboratories (63%) expressed willingness to implement these fields into their laboratory requisition forms, and the remaining 34% qualified their response, because the decision would be subject to review by a clinical expert, information technology team, and/or corporate approval.

### ClinGen Expert Panel Survey and Final Consensus

Of 19 entities represented in the expert panel, 14 participated. The consensus group, formed by laboratory directors, cardiologists, and genetic counselors, representing commercial and academic institutions in the United States and abroad, provided full endorsement of the following recommendations: i) there should be consensus on which data elements should be standard fields on requisition forms; ii) the expert panel considers the minimal phenotypic data elements as critical; iii) these data should be provided by clinicians when ordering genetic testing. Consistent with previous observations in the LMM pilot study and Laboratory Director Survey, some participants shared that blood pressure data would be difficult to obtain and thus also recommended against these data being part of the essential criteria for variant classification. The expert panel survey further reduced the list to nine data elements that were deemed essential for HCM variant classification. The results of this survey were presented during a conference call with the expert panel, during which the group affirmed consensus.

This consensus process resulted in a Laboratory Requisition Module that could be implemented to optimize collection of phenotypic data for HCM on requisition forms (Table 3). This module uses a tiered approach, with the nine data elements deemed critical for counting and characterizing cases for variant classification presented first, followed by those representing the phenotypic data elements that were considered nonessential by the expert panel. Because these data are critical for accurate case counts during variant interpretation, the ClinGen Expert Panel proposes that ordering providers routinely provide these essential data elements and that laboratories reach out to providers to obtain these data.

**Table 3:** Laboratory Requisition Module for HCM

Essential elements	Field type	Format or options
Birth sex	Selection	Male, female, other, or unknown
Race and ethnicity∗	Selection	American Indian or Alaska Native, Asian, Black or African, Native Hawaiian or other Pacific Islander, White, or Hispanic
Current age	Free text	Years, or months if <1 year old
Family history	Selection and free text	None, unknown, HCM, left ventricular hypertrophy, cardiomyopathy, sudden cardiac death, or other (free text)
Clinical diagnosis of HCM,† HPO,‡ HP:0001639, or MONDO:0005045§	Selection	Yes, no, or unknown
Age at diagnosis	Free text	Years, or months if <1 year old
Left ventricular hypertrophy (HPO HP:0001712)	Selection	Yes, no, or unknown
Maximum left ventricular wall thickness	Free text	Centimeters or millimeters
Suspected syndromic HCM/other cause	Selection and free text	Fabry disease, Danon disease, skeletal muscle weakness, or other (free text)
Nonessential elements		
Left ventricular outflow tract obstruction	Selection	Yes, no, or unknown
Reduced ejection fraction (HPO HP:0012664)	Selection	Yes, no, or unknown
Left ventricular ejection fraction (in %)	Free text	Percentage
History of hypertension (HPO HP:0000822)	Selection	Yes, no, or unknown
Blood pressure on treatment	Free text	Systolic/diastolic blood pressure
ECG with left ventricular hypertrophy (HPO HP:0001712) or atrial fibrillation (HPO HP:0005110)	Selection	Yes (if yes: specify), no, or unknown
History of syncope (HPO HP:0001279)	Selection	Yes, no, or unknown
Nonsustained ventricular tachycardia on Holter	Selection	Yes, no, or unknown
Late gadolinium enhancement on cardiac MRI	Selection	Yes, no, or unknown

ECG, electrocardiogram; HCM, hypertrophic cardiomyopathy; HPO, Human Phenotype Ontology; MONDO, Mondo Disease Ontology; MRI, magnetic resonance imaging.

∗ Racial and Ethnic Categories and Definitions for NIH Diversity Programs and for Other Reporting Purposes (*https://grants.nih.gov/grants/guide/notice-files/not-od-15-089.html*, last accessed October 22, 2020).

† Fulfilling criteria for the clinical diagnosis of HCM, per ordering clinician's assessment.

‡ HPO, (*hpo.hax.org*, last accessed November 4, 2020).

§ MONDO, (*https://monarchinitiative.org*, last accessed November 4, 2020).

## Discussion

This article presents a minimum set of clinical data elements that should be used for variant classification in the context of genetic testing for HCM. Support and acceptance of this nine-element data set across participating laboratories were high, with few modifications to the original expert panel proposed criteria. This harmonization effort was intended to aid interpretation and simplify requisition forms to improve ordering provider compliance. The successful implementation of this effort will produce a substantial amount of aggregate data that should, in turn, be standardized with data collection efforts from public databases, such as ClinVar. The implementation of acquiring this lean data set for each patient resides with the clinical testing laboratories; however, its success relies on the ordering clinicians' willingness to provide the minimum phenotypic data set. Moreover, provider collaboration is more likely with wide adoption of this data set among laboratories. In fact, although receiving results with variants classified as variants of uncertain significance remains a major reason for frustration for health care providers, many tests are ordered without providing even the limited information requested by the laboratories. Having a minimal data set, such as the one proposed in this study, could ensure a subject meets criteria for the aforementioned American College of Medical Genetics and Genomics/Association for Molecular Pathology guidelines, potentially leading to a clinically significant variant reclassification that could incentivize ordering providers to submit relevant clinical information.

The chance of success in obtaining key data for variant interpretation could be improved by reinforcing efforts using a three-pronged approach, addressing gaps in clinic, education, and research. Cardiologists can benefit from increased collaboration with genetics providers to enhance and improve clinical care with genotype-informed management. Empirical observation suggests that genetics providers are more likely to include relevant clinical data in requisition forms that could enable efficient collaboration with clinical laboratories in this area. Ordering providers can also benefit from practical education on variant interpretation to illustrate the utility of appropriate case data when evaluating evidence for a given variant. Finally, studies that could shed light on the barriers impeding clinical data sharing and that show the value of this work are necessary. Ultimately, providers will be incentivized to provide relevant data when the process is simplified and made relevant by reducing uncertain variant classifications.

### Evaluation of Phenotypic Data Elements

Several of the proposed phenotypic data elements (eg, sex and race and ethnicity) of the proposed HCM phenotypic data set are commonly included on laboratory requisition forms and routinely provided to the laboratory by the ordering clinicians in current practice, affirming their importance. Age of onset and LVH measurement are also critical data elements that are less frequently collected; however, they were deemed essential for variant interpretation. Although data from the LMM pilot support that most of the phenotypic data elements on requisition forms are not routinely completed by ordering providers, it is possible that a sole entry of a clinical diagnosis of HCM, which was determined to be present in 98% of the evaluated cases, may have resulted in exclusion of some of the other critical data elements. This practice of providing limited phenotypic data is at the core of this work, as excluding the other data elements, some of which are essential for identifying a countable case, would preclude a laboratory from classifying an otherwise rare and poorly understood variant.

Collecting additional information carries the potential for improved characterization of genotype-phenotype relationships, as exemplified by variants in the thin-filament genes *TNNT2*, *TNNI3*, *TPM1*, and *ACTC1*, which can be associated with milder and atypically distributed LVH.^15^ In addition, phenotypic data not typically associated with primary HCM are important in helping to determine whether LVH may be secondary to a different process. For example, a diagnosis of HCM in the context of an extensive history of athletic training or severe, uncontrolled hypertension requires special consideration when assessing the pathogenicity of a variant. Other phenotypic data elements that were deemed not essential for variant interpretation (left ventricular outflow tract obstruction, reduced ejection fraction and percentage, electrocardiogram with LVH or atrial fibrillation, history of syncope, nonsustained ventricular tachycardia on Holter monitoring, and late gadolinium enhancement on cardiac magnetic resonance imaging) may be less frequently obtained owing to an absence of fields for these data on requisition forms. However, during review of the proposed minimal phenotypic data elements, all study participants (including the laboratories that currently collect this information) were in favor of not categorizing these as essential as they were not anticipated to provide useful data.

### Practical Considerations and Acceptance of Recommendations

It is expected that these criteria will be implemented by the participating laboratories and provide further impetus for additional guidelines and refinement of phenotypic data elements. The ultimate power of standardizing data collection across laboratories lies in the ability to harness aggregate data, provided that all laboratories share their data into public databases that are structured to accommodate such standardized data. This illustrates a timely intersection between clinical genetic testing and academic consortium research efforts, providing fertile ground for collaboration. With its mission of building a genomic knowledge base to improve patient care and its guidance of expert variant curation, ClinGen is ideally situated to fulfill this role. Following recognition by the Food and Drug Administration, ClinGen has tasked variant curation expert panels with reassessing variant classifications in ClinVar. The data collected from harmonized laboratory requisitions could serve as a foundation for this process, particularly if ClinVar supports the collection of phenotypic data elements essential for variant classification along with an algorithm that aggregates proband counts across laboratories.

This study has some limitations. First, because of the small number of laboratories participating in the survey, there remains a possibility of ascertainment bias. These laboratories were recruited because they offer cardiomyopathy genetic testing and used expert panel members or associates. At the same time, although it is acknowledged that the participating laboratories are a highly selected group, they represent the vast majority of the cardiovascular cardiomyopathy genetic testing volume in the United States. Second, the Laboratory Directors Survey asked participants to select the frequency in which the data elements were provided by ordering providers to later inform the decision of whether a data element would be excluded from the final consensus list. As the participants were not asked to run a formal case review to provide a percentage of completion of each data element, their selection (very frequently/frequently/sometimes/very infrequently/infrequently) may have involved a degree of subjectivity in defining the various categories. Notwithstanding, this assessment was vetted by practicing cardiologists who are members of the expert panel who agreed that some of these items are not universally obtained for every patient with HCM. For example, not every patient with HCM will present with left ventricular outflow tract obstruction or have cardiac magnetic resonance imaging for late gadolinium enhancement assessment.

It should be recognized that the essential data elements are a limited list that can nonetheless serve as a starting point for laboratories to develop additional fields to capture other additional data as needed. Similarly, as a proof of concept, HCM was chosen for this study, but the application of standardizing the minimum data elements essential for variant classification should not be limited to HCM. Future directions include a follow-up study consisting of case evaluations before and after consensus to demonstrate the utility of laboratory requisition form harmonization by, for example, showing a reduction in variant of uncertain significance classifications. In addition, similar processes expanding the minimum phenotypic data set for case-level data in other cardiovascular phenotypes and in rare diseases, where the availability of detailed phenotype data has enabled new diagnoses, would be important contributions to the field. The results of future studies like these could be integrated into guidelines providing disease and gene specifications for variant classification.

Beyond application and implementation of additional guidelines, data sharing remains a powerful tool for genetic analysis and variant resolution. A proposed future application of the recommendations presented herein is to increase the granularity of laboratory submissions to ClinVar. ClinVar provides an opportunity to improve the accuracy of classified variants by capturing phenotypic data elements across the referral base for genetic testing. Cohesion between ClinVar, laboratories, and ordering providers is a highly desired strategic goal for harmonization of gene and sequence variant classifications.

## Conclusion

The fields intended to collect phenotypic data on requisition forms for HCM genetic testing are widely divergent across laboratories, and the data elements important for variant classification are not uniformly collected. This study attempts to close this gap by defining the minimum data elements that are both useful and critical for counting and characterizing cases during variant classification for HCM. Endorsement of these phenotypic data elements can aid when counting cases for variant classification but does not substitute for HCM clinical diagnostic criteria or signify that clinical testing should be delayed or prevented in the absence of these data. This process could be replicated for use in other genetic conditions and could be leveraged to enhance the utility of ClinVar. Ultimately, fulfilling the potential of this concept requires a collaborative relationship between the laboratories and ordering providers, one marked by a commitment to data sharing and high-quality care.
