# Supplementary material for: Harmonizing the Collection of Clinical Data on Genetic Testing Requisition Forms to Enhance Variant Interpretation in Hypertrophic Cardiomyopathy (HCM): A Study from the ClinGen Cardiomyopathy Variant Curation Expert Panel
Source: J Mol Diagn. 2021 May;23(5):589–98. doi: 10.1016/j.jmoldx.2021.01.014 (PMC8188618; doi:10.1016/j.jmoldx.2021.01.014)
Supplement: Supplemental Appendix S1 [file mmc1.pdf]

Supplementary Appendix S1
ClinGen Lab Requisition Harmonization: CM-EP Lab Directors Survey

ClinGen Lab Requisition Harmonization: CM-EP Lab Directors Survey

.

ClinGen Lab Requisition Harmonization: CM-EP Lab Directors Survey

Q1. The list below contains clinical information deemed to be essential to the diagnosis of HCM, as determined by the ClinGen Cardiovascular Disease Working Group (ClinGen Phenotypic Data Elements List).

Do you agree these constitute the minimal phenotypic data useful for HCM variant classification in **probands**?

Sex
Ethnicity
Current Age
Family History

Clinical diagnosis of HCM
Age at diagnosis

Left Ventricular Hypertrophy
Left Ventricular Hypertrophy Measurement
Left Ventricular Outflow Tract (LVOT) Obstruction
Reduced Ejection Fraction
Reduced Ejection Fraction Percentage

History of Hypertension
Blood Pressure on Treatment
Suspected Syndromic HCM/Other Cause
ECG with Left Ventricular Hypertrophy (LVH) or Atrial Fibrillation (AF)

History of Syncope

Non Sustained Ventricular Tachycardia (NSVT) on Holter

Late Gadolinium Enhancement (LGE) on CMR

Yes, I agree with these criteria as essential information

No, I disagree with these criteria as essential information

Yes, I agree with these criteria as essential information, but feel it is incomplete (please comment)

Other (please comment)

Q2.

Do you agree these constitute the minimal phenotypic data useful for HCM variant classification in family members of probands with HCM?

Sex

Ethnicity

Current Age

Family History

Clinical diagnosis of HCM

Age at diagnosis

Left Ventricular Hypertrophy

Left Ventricular Hypertrophy Measurement

Left Ventricular Outflow Tract (LVOT) Obstruction

Reduced Ejection Fraction

Reduced Ejection Fraction Percentage

History of Hypertension

Blood Pressure on Treatment

Suspected Syndromic HCM/Other Cause

ECG with Left Ventricular Hypertrophy (LVH) or Atrial Fibrillation (AF)

History of Syncope

Non Sustained Ventricular Tachycardia (NSVT) on Holter

Late Gadolinium Enhancement (LGE) on CMR

Yes, I agree with these criteria as essential information

No, I disagree with these criteria as essential information

Yes, I agree with these criteria as essential information, but feel it is incomplete (please comment)

Other (please comment)

Q3. Would you be willing to implement these fields into your laboratory's requisition form?

Yes (please comment)

Maybe (please comment)

No (please comment)

Q4. Based on the ClinGen Phenotypic Data Elements List, how frequently do you obtain the clinical data that you need for variant classification?

	Very Frequently	Frequently	Sometimes	Infrequently	Very Infrequently
Sex	○	○	○	○	○
Ethnicity	○	○	○	○	○
Current Age	○	○	○	○	○
Family History	○	○	○	○	○
Clinical Diagnosis of HCM	○	○	○	○	○
Age at Diagnosis	○	○	○	○	○
Left Ventricular Hypertrophy	○	○	○	○	○

	Very Frequently	Frequently	Sometimes	Infrequently	Very Infrequently
Left Ventricular Hypertrophy Measurement	○	○	○	○	○
Left Ventricular Outflow Tract (LVOT) Obstruction	○	○	○	○	○
Reduced Ejection Fraction	○	○	○	○	○
Reduced Ejection Fraction Percentage	○	○	○	○	○
History of Hypertension	○	○	○	○	○
Blood Pressure on Treatment	○	○	○	○	○
Suspected Syndromic HCM/Other Cause	○	○	○	○	○
ECG with Left Ventricular Hypertrophy (LVH) or Atrial Fibrillation (AF)	○	○	○	○	○
History of Syncope	○	○	○	○	○
Non Sustained Ventricular Tachycardia (NSVT) on Holter	○	○	○	○	○
Late Gadolinium Enhancement (LGE) on CMR	○	○	○	○	○

Q5. How often do you rely on clinical data for variant classification?

Very Frequently

Frequently

Sometimes

Infrequently

Very Infrequently

Q6.
For the purpose of variant assessment, how often do you have to contact the ordering provider for additional clinical information?

Very Frequently

Frequently

Sometimes

Infrequently

Very Infrequently

Never

Q7. Which of the following findings would suggest you or your staff to call the ordering provider for additional information? (select all that apply)

Discrepant phenotype

Syndromic phenotype

Incomplete phenotype

Other (please comment)

Q8. At what point is clinical data the *most* critical for your interpretation?

Prior to variant assessment

During variant assessment

After variant assessment

Other (please comment)

Q9. How specialized do you consider your lab to be in cardiomyopathy?

Very specialized

Specialized

Neutral

Not Specialized

Not Specialized At All

Q10. For a single report, approximately how many staff evaluate a cardiomyopathy result?

1-4

5-9

10-15

15+

Q11. Does your laboratory have a specialized team for cardiomyopathy?

Yes

No

Q12. Does your laboratory have pre-existing essential clinical criteria for each case (i.e. minimal criteria met that could define a variant identified in a "true" case vs. phenocopy)?

Yes

No

[ ] Somewhat (comment)

Q13. What are the roles of your staff members that work on cardiomyopathy reports?

	Case and requisition review	Variant Assessment	Report Drafting	Report Review	Report Signout	Lab-Provider Communication	ap
MD	☐	☐	☐	☐	☐	☐	
PhD	☐	☐	☐	☐	☐	☐	
MD/PhD	☐	☐	☐	☐	☐	☐	
MS CGC, Genetic Counselor	☐	☐	☐	☐	☐	☐	
MS	☐	☐	☐	☐	☐	☐	
BS/BA	☐	☐	☐	☐	☐	☐	
Other (please comment) [ ]	☐	☐	☐	☐	☐	☐	

Q14. Are there any additional thoughts or comments about this project that you wish to share?
