# Supplementary material for: Harmonizing the Collection of Clinical Data on Genetic Testing Requisition Forms to Enhance Variant Interpretation in Hypertrophic Cardiomyopathy (HCM): A Study from the ClinGen Cardiomyopathy Variant Curation Expert Panel
Source: J Mol Diagn. 2021 May;23(5):589–98. doi: 10.1016/j.jmoldx.2021.01.014 (PMC8188618; doi:10.1016/j.jmoldx.2021.01.014)
Supplement: Supplemental Appendix S2 [file mmc2.pdf]

Supplementary Appendix S2

ClinGen: Harmonizing Molecular Diagnostic Testing

Clinical Data Collection Working Group Survey

(ClinGen Expert Panel Survey)

Default Question Block

ClinGen: Harmonizing Molecular Diagnostic Testing Clinical Data Collection Working Group Survey

Name:

[ ]

Do you endorse the recommendation that there should be specific minimal consensus criteria that should be present as standard fields on laboratory requisition forms?

- ○ Yes, I agree- there are specific minimal consensus criteria fields that should be present as standard on laboratory requisition forms
- ○ No, I disagree- there are NOT specific minimal consensus criteria fields that should be present as standard on laboratory requisition forms
- ○ Yes, I agree there are specific minimum consensus criteria fields that should be present as standard on laboratory requisition forms, but have additional feedback (please comment): [ ]
- ○ Other (please comment): [ ]

Do you endorse the recommendation that ordering providers should consistently provide this information (i.e. clinical data related to established minimal consensus criteria) to laboratories for patients receiving genetic testing for HCM as standard practice?

- ○ Yes, I agree- clinicians should consistently attempt to provide this information (clinical data related to specific minimal consensus criteria) as standard practice
- ○ No, I disagree- clinicians should NOT consistently attempt to provide this information (clinical data related to specific minimal consensus criteria) as standard practice
- ○ Yes, I agree – clinicians should consistently attempt to provide this information (clinical data related to specific minimal consensus criteria) as standard practice, but have additional feedback (please comment): [ ]
- ○ Other (please comment): [ ]

These criteria represent the minimum key clinical data elements proposed by lab directors as recommendations for standard inclusion for variant interpretation in HCM genetic testing. Please indicate your endorsement or disapproval for each criteria below.

	Endorse	Do Not Endorse
Sex	○	○
Ethnicity	○	○
Current Age	○	○
Family History	○	○
Clinical Diagnosis of HCM	○	○
Age at Diagnosis	○	○
Left Ventricular Hypertrophy	○	○
Left Ventricular Hypertrophy Measurement	○	○
History of Hypertension	○	○
Blood Pressure on Treatment	○	○
Suspected Syndromic HCM/other cause	○	○

These criteria are only intended to reflect recommendations for HCM. For publication and longitudinal purposes, do you feel that:

- ○ Recommendations for HCM should be submitted as a stand-alone
- ○ Recommendations for additional cardiomyopathies (e.g. DCM and ARVC) be developed and submitted with HCM as part of a larger recommendation

A proposed future application of these recommendations is to increase the granularity of laboratory submissions to ClinVar. If adoption of these criteria is successful from both the laboratory and clinical perspective, do you endorse a proposal for the addition of fields from the data elements list approved by this panel to ClinVar entries? (e.g. LVH measurement)

- ◯ Yes, endorse proposal for addition of select fields to ClinVar
- ◯ No, do not endorse proposal for addition of select fields to ClinVar

Please provide any additional feedback not otherwise captured in this document

Powered by Qualtrics
